# The Synchronization Transceiver Design and Experimental Verification for the LuTan-1 SAR Satellite

**DOI:** 10.3390/s20051463

**Published:** 2020-03-06

**Authors:** Yuanbo Jiao, Da Liang, Kaiyu Liu, Yafeng Chen, Huaizu Wang, Robert Wang

**Affiliations:** 1 Space Microwave Remote Sensing System Department, Aerospace Information Research Institute, Chinese Academy of Sciences, Beijing 100190, China; jiaoyb@aircas.ac.cn (Y.J.); liangda16@mails.ucas.edu.cn (D.L.); chenyf@aircas.ac.cn (Y.C.); wanghz@aircas.ac.cn (H.W.); yuwang@mail.ie.ac.cn (R.W.); 2 School of Electronic, Electrical and Communication Engineering, University of Chinese Academy of Sciences, Beijing 100039, China

**Keywords:** bistatic synthetic aperture radar (BiSAR), phase synchronization, non-interrupted, synchronization transceiver (STR)

## Abstract

The deviation between the two oscillators in BiSAR systems will cause a residual modulation of echo signal. Therefore, the phase synchronization is an important issue that must be addressed for BiSAR systems. An advanced non-interrupted phase synchronization scheme is used for the LuTan-1 SAR satellite. The synchronization transceiver (STR) is designed for transmitting and receiving synchronization signals. In addition, STR mainly consists of master and auxiliary transceivers and switch module. Furthermore, the function and working principle of STR are introduced, and the detailed design of each part is described. The measured results are also evaluated to prove the performance of the STR. In addition, the phase synchronization accuracy is also demonstrated to verify the effectiveness of the non-interrupted synchronization scheme. The standard deviation (STD) of the residual phase is less than 0.3 degrees. The results have guiding significance for the synchronization unit design of LuTan-1 and the future BiSAR system.

## 1. Introduction

Synthetic aperture radar (SAR) is one of the most important tools for earth observation [1]. Bistatic SAR (BiSAR) operates with distinct transmitters and receivers, which attracts with increasing attention due to its advantages [2,3]. The spaceborne BiSAR can be used for generating digital elevation models (DEM) of global earth [4]. The BiSAR can also be applied in monitor, topography cartography, deformation detection, agriculture, classification, and so on [3,5,6]. In recent years, some new spaceborne bistatic and multistiatc SAR proposals are proposed, such as TanDEM-L [7], SAOCOM-CS [8], SESAME [9], MirrorSAR [10], and Harmony [11].

The advantages of BiSAR system are accompanied by new challenges [12]. One of the most challenging problems is the phase synchronization for BiSAR systems [13,14,15,16,17,18]. In BiSAR systems, the transmitter and receiver use different oscillators. Therefore, there will be a frequency offset between the two oscillators, which will cause a residual modulation in the SAR raw data. Many phase synchronization methods have been proposed in the last few decades. In a continuous duplex synchronization scheme, the primary system and slave system both continuously transmit the synchronization signal during the data take [19]. The pulsed duplex synchronization and the pulsed alternate synchronization schemes were also proposed in [20]. In addition, the pulsed alternate synchronization scheme is used in TanDEM-X. Due to the interruption of normal SAR operation, the data reconstruction is essential for TanDEM-X [21].

As an important part of the medium- and long- term development plan for China’s civil space infrastructure, LuTan-1 mission was formally established in 2016 and will be launched in 2021 [22]. It mainly focuses on differential interferometry, deformation measurement, and full-polarization interferometry for global earth. It is based on the use of two cooperative SAR satellites with flexible formation flying. In LuTan-1 systems, an innovative non-interrupted synchronization scheme is used which will not interrupt the normal SAR operation [23]. LuTan-1 exchanges the synchronization signals immediately after the ending time of the radar echo-receiving windows and before the starting time of the next pulse-repetition internal (PRI) [18,24]. Therefore, it cannot interrupt the normal SAR data acquisition, further improving the synchronization accuracy and avoiding the data missing effect.

In order to achieve the phase synchronization for LuTan-1, a novel synchronization transceiver (STR) is developed for the first time in the central electronics subsystem. The STR is used for transmitting and receiving the synchronization signal [25]. The function and working principle of STR are described in the paper. The structure of STR is introduced in detail. The STR contains master transceiver, auxiliary transceiver, and switch module. Each transceiver contains a T-branch, R-branch, and common branch [26]. The performance of each part is analyzed. The measured results are also evaluated to prove the performance of the STR. The standard deviation (STD) of the residual phase is less than 0.3 degrees, which proves that the synchronization scheme has high synchronization accuracy.

This paper is organized as follows. The non-interrupted phase synchronization scheme is introduced in Section 2. The function and working principle of STR are introduced in Section 3. The design of the STR is described in Section 4. In Section 5, the test results are used to verify the effectiveness of STR. Section 6 concludes the paper.

## 2. Phase Synchronization Scheme of LuTan-1

LuTan-1 mission is an innovative spaceborne BiSAR system in L-band. Its main mission is to acquire high accurate DEM and support some civil applications, such as biomass inversion, disaster forecasting, and climate and environmental monitoring [22]. The GNSS disciplining module in LuTan-1 mission employs a disciplined rubidium clock to provide a time-frequency signal for the SAR systems with high accuracy [24,27,28,29,30]. However, the oscillator noise between the two satellites will severely degrade the performance of the LuTan-1 imaging and interference processing [13]. Therefore, the phase synchronization is an important issue that must be addressed for BiSAR systems. In this section, the synchronization system model is established. The non-interrupt synchronization scheme of LuTan-1 is also described in detail.

### 2.1. Synchronization System Model

**Figure 1:**
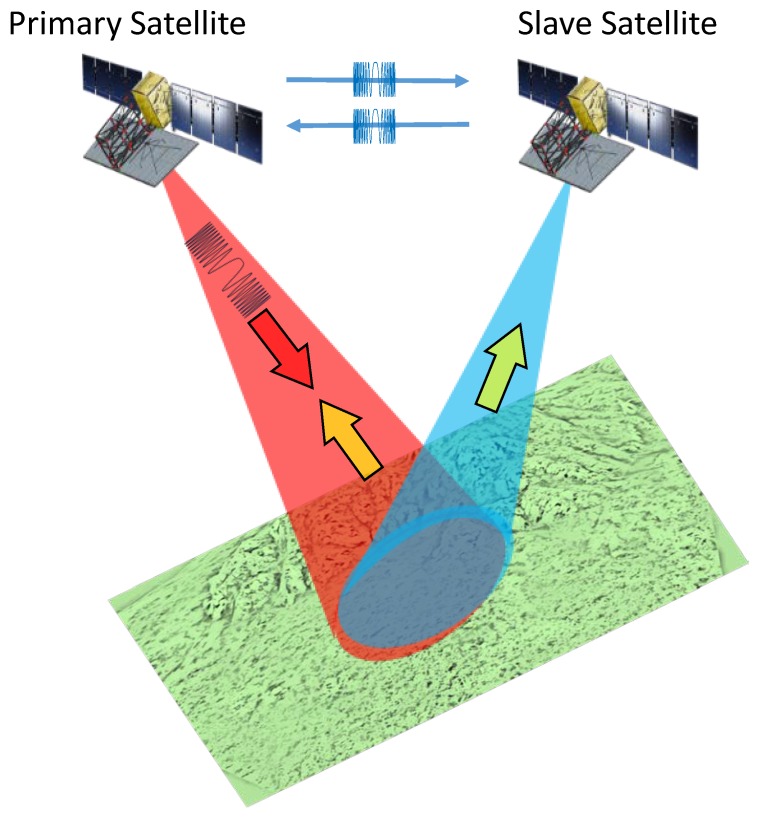
Artist’s view of the LuTan-1 mission.

As shown in Figure 1, the LuTan-1 mission is based on the use of two cooperative SAR satellites. The primary satellite transmits the radar signal to the ground. Both satellites receive the echoes from the ground. Suppose the frequency of the primary satellite is $f_{1}\left(t\right)$ and the frequency of the slave satellite is $f_{2}\left(t\right)$ at time *t*. Thus, the phases of two satellites can be written as follows [14,23]:

(1) $$\begin{array}{cc} \varphi_{1}\left(t\right) & =2\pi f_{1}\left(t\right)t+n_{\varphi_{1}}\left(t\right) \end{array}$$

(2) $$\begin{array}{cc} \varphi_{2}\left(t\right) & =2\pi f_{2}\left(t\right)t+n_{\varphi_{2}}\left(t\right) \end{array}$$

where $n_{\varphi_{1}}\left(t\right)$ and $n_{\varphi_{2}}\left(t\right)$ denote the phase noise in primary satellite and slave satellite, respectively. After an echo delay $\tau_{R}$, the transmitted radar signal by primary satellite is reflected back to the slave satellite. Therefore, the phase in slave satellite after demodulation can be represented as

(3) $$\begin{array}{cc} \Delta \varphi_{21}\left(t\right) & =\varphi_{1}\left(t-\tau_{R}\right)-\varphi_{2}\left(t\right)\\ \; & =-2\pi f_{1}\left(t\right)\tau_{R}-2\pi \left(f_{2}\left(t\right)-f_{1}\left(t\right)\right)t+n_{\varphi_{1}}\left(t-\tau_{R}\right)-n_{\varphi_{2}}\left(t\right) \end{array}$$

The first term in Equation (3) is useful signal for SAR imaging. The second term in Equation (3) denotes the frequency offset between the two satellites, which should be corrected in BiSAR processing. The phase synchronization is designed to extract the frequency offset, which is essential for BiSAR systems.

In order to achieve phase synchronization, a common phase reference needs to be established between the primary and slave satellite [24]. For this purpose, it is considered to establish a phase synchronization link between the two satellites. As shown in Figure 1, the synchronization signals are exchanged between the two satellites.

The demodulated phase $\varphi_{12}\left(t\right)$ is available at the primary satellite for a synchronization signal transmitted by the slave satellite. Correspondingly, the demodulated phase $\varphi_{21}\left(t\right)$ is available at a slave satellite for a synchronization signal transmitted by a primary satellite [24]. In essence, the synchronization signal can be regarded as the special radar signal. The synchronization signal and radar signal all include the frequency information of oscillators. However, the synchronization signal only represents the signal of one target, which is different from the radar echoes, represented the signals of area target. Therefore, after pulse compression of a synchronization signal, the demodulated phase $\varphi_{12}\left(t\right)$ and $\varphi_{21}\left(t\right)$ can be obtained by extracting peak phase. Suppose τ denotes the synchronization signal travel time between the primary satellite and slave satellite. $\varphi_{12}\left(t\right)$ and $\varphi_{21}\left(t\right)$ all contain the phase terms caused by the travel time τ. Therefore, the phase term caused by the travel time τ can be eliminated by $\varphi_{21}\left(t\right)-\varphi_{12}\left(t\right)$. The effect of travel time differences between the two transmissions of synchronization signal can be eliminated by Doppler phase $\varphi_{dop}$. As a result, the compensate phase $\varphi_{c}\left(t\right)$ can be expressed as [14,23]

(4) $$\begin{array}{cc} \varphi_{c}\left(t\right) & =\frac{1}{2}\left(\varphi_{21}\left(t\right)-\varphi_{12}\left(t\right)\right)\\ \; & \approx 2\pi \left(f_{1}\left(t\right)-f_{2}\left(t\right)\right)t+\pi \left(f_{1}\left(t\right)-f_{2}\left(t\right)\right)\left(\tau +\tau_{sys}\right)+\varphi_{dop}+\varphi_{res} \end{array}$$

where $\varphi_{res}$ denotes the other phase errors, such as oscillator noise phase error and hardware system phase error [18]. The transmit instance of slave satellite is delayed by $\tau_{sys}$ with respect to primary satellite. $\varphi_{dop}$ can be expressed as [14]

(5) $$\varphi_{dop}=-\pi f_{D}\tau_{sys}$$

where $f_{D}$ is the Doppler frequency due to the relative velocity between the two satellites. $\varphi_{dop}$ should also be corrected according to the relative motion between the two satellites.

### 2.2. Non-Interrupted Synchronization Scheme for LuTan-1

**Figure 2:**
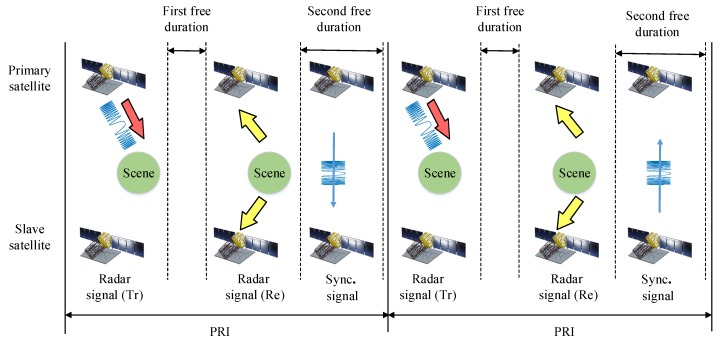
Timing diagram of synchronization signal exchange.

In order to exchange the exchange the synchronization pulse, an accurate timing diagram of the synchronization signal exchange is designed for LuTan-1, which prevents the interruptions of the normal SAR operation. The timing diagram of the synchronization signal exchange is shown in Figure 2. In one PRI, there are two time windows used for radar system. The first time window is used for transmitting radar signals. Correspondingly, the second time windows are used for receiving the echoes from the ground. As a result, there are two free durations. As shown in Figure 2, the first free duration is the free duration between the time of the end of the radar signal transmission and the starting time of the echo receiving windows, and, correspondingly, the second free duration is the free duration between the time of the end of echo receiving window and the starting time of the next PRI [23]. In the LuTan-1 system, the phase synchronization signal is exchanged in the second free duration [24]. Thus, the normal operation of SAR can be prevented from being interrupted, avoiding the data missing effect [18].

**Figure 3:**
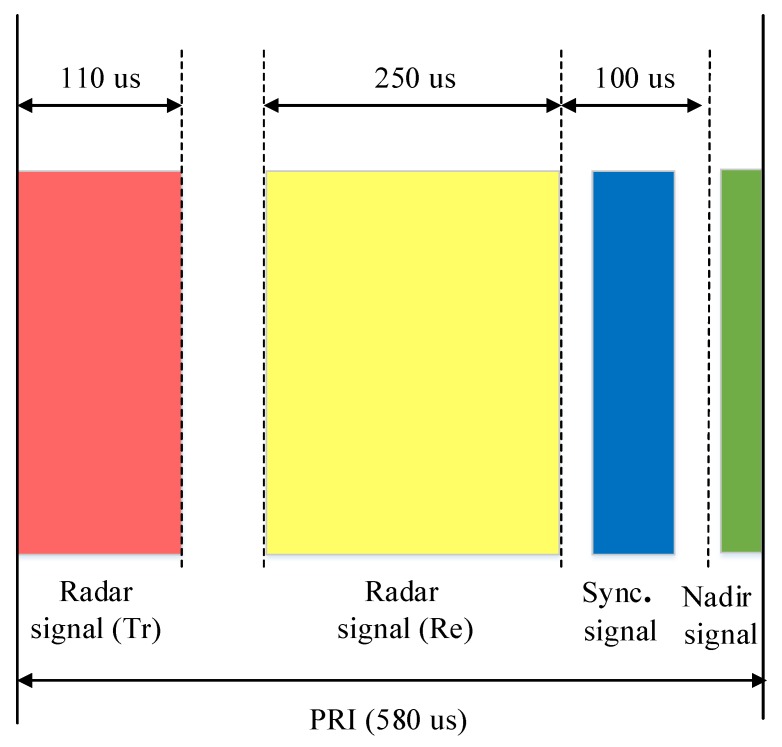
Timing diagram of the LuTan-1 mission.

The main parameters of LuTan-1 mission are shown in Table 1. The maximum value of the synchronization rate can be set to the half of the pulse-repetition frequency (PRF). An example of beams for the LuTan-1 mission is shown to illustrate the non-interrupt synchronization scheme. The main parameters of example are shown in Table 2. The timing diagram is shown in Figure 3. The synchronization pulse width is set to 10 μs, which is sufficient to meet the signal-to-noise ratio (SNR) requirement of synchronization. The pulse-repetition frequency is 1723 Hz and PRI is 580 μs. The pulse width of the radar signal is 110 μs and the echo receiving window width is 250 μs. In the second free duration, there are 115.6 us of free time. The baseline between the two satellites is less than 10 km. Therefore, the synchronization pulse exchange should be no more than 53 μs. The effect of nadir echo should also be considered. From Figure 3, it can be seen that the spare time for synchronization pulse exchange is more than 100 μs, which is enough for synchronization pulse exchanging.

## 3. Function and Working Principle of Synchronization Transceiver

In order to achieve the phase synchronization for LuTan-1, a novel STR is developed for the first time in the electronics subsystem. The synchronization signal is transmitted and received by the STR. In this section, the function and working principle of STR are introduced in detail.

**Figure 4:**
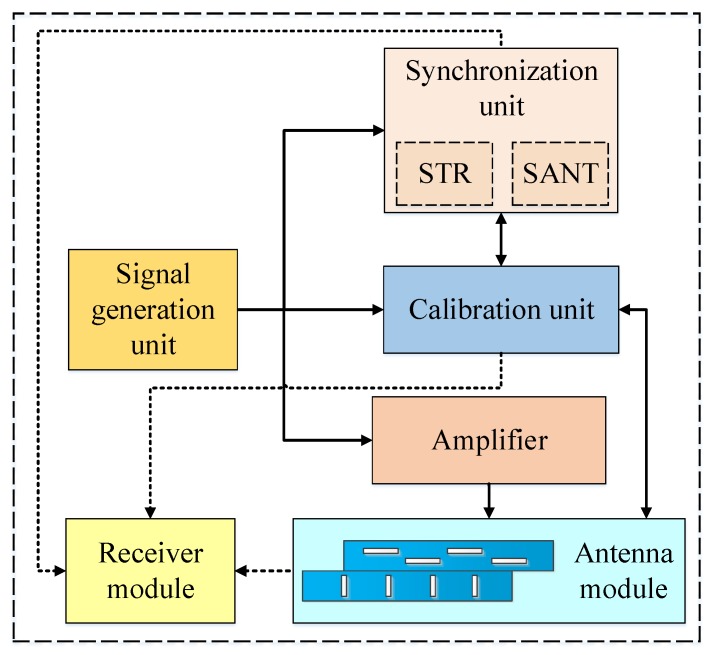
The structure block diagram of the LuTan-1 system.

**Figure 5:**
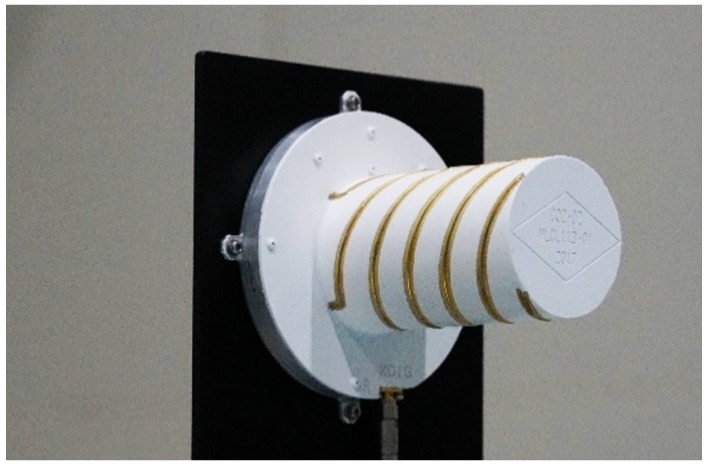
Photograph of a quadrifilar helix antenna.

The hardware structure diagram of the LuTan-1 system is illustrated in Figure 4. The system consists of the signal generation unit, the transmit–receive unit, the synchronization unit, and the calibration unit. The synchronization unit is mainly composed of STR and synchronization antenna (SANT). In the LuTan-1 system, the quadrifilar helix antenna is used for SANT, which can be seen in Figure 5. The quadrifilar helix antenna has a wide beam. In order to achieve omnidirectional coverage during an orbital period, both primary and slave satellites are equipped with four synchronization antennas.

**Figure 6:**
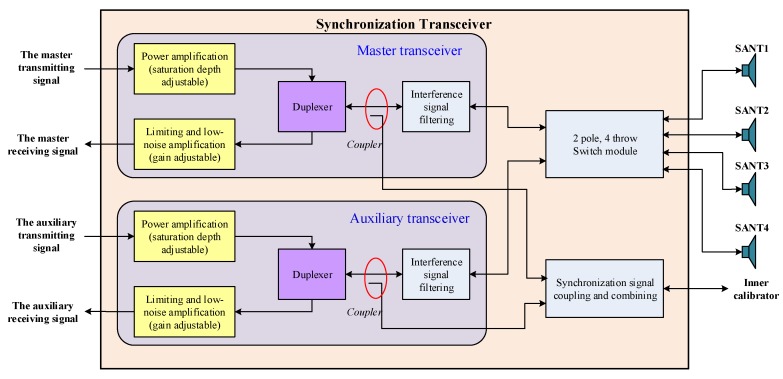
The schematic diagram of STR.

**Figure 7:**
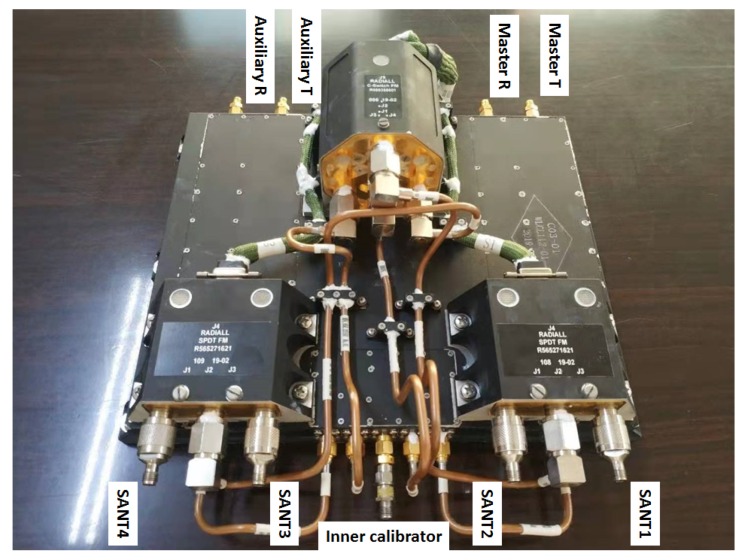
The physical photo of STR.

STR is mainly composed of master transceiver, auxiliary transceiver, microwave switches, and so on. The master and auxiliary transceiver are isolated from each other and have the same circuit principle. In general, the master transceiver is used in the system. However, if the master transceiver is broken, the auxiliary transceiver can be used for transmitting and receiving synchronization signals. This design significantly improves the reliability of STR. The circuit topology of the STR includes power amplifier (PA), circulator, switches, low-pass filter (LPF), limiter, directional coupler, and low-noise amplifier (LNA). The schematic diagram of the STR is shown in Figure 6. The physical photo of the STR is shown in Figure 7. In the transmitting mode (T-mode), the synchronization signal is transmitted through the STR and SANT. In the receiving mode (R-mode), the synchronization signal is received by the SANT, and then it is routed through the STR and microwave combination. Finally, the synchronization signal is demodulated and recorded by the receiver; then, it is processed by the former data. The recorded data of synchronization signals are sent to the ground processing system by the data transmission system.

The STR should have the following functions: receiving and transmitting synchronization signals separately, switching between the master and auxiliary transceiver and different SANT ports [31]. The specific functional principles are as follows:

1. Power amplification of the synchronization signal by the master/auxiliary transceiver.
2. Switch to a certain SANT according to the control signal from the Control and Timing unit.
3. Amplitude limiting and low-noise amplification of the received synchronization signals.
4. Obtain effective spatial interference suppression of synchronization signals.
5. Switch between the master and auxiliary transceiver link.
6. Couple synchronization signal and generate calibration signal.

In order to achieve good phase synchronization between two satellites, the design of the STR shall take into account broadband, low noise, linear phase response, sufficient transmitting power, receiving gain, etc. The main technical requirements of the STR are shown in Table 3. The operation bandwidth should be larger than 150 MHz, which corresponds to the maximum bandwidth of radar signal. The sufficient transmitting power and receiving gain are designed to ensure the received synchronization signal with high SNR. The in-band flatness of amplitude and nonlinear phase error of transmitting and receiving signal, noise figure (NF), and gain of receiving signal and isolation of switches are with the high standard requirement, which can effectively reduce the influence on the synchronization accuracy. The power consumption, weight, and size need to be properly considered to occupy less resources on the satellite.

## 4. Design of STR

The STR contains master transceiver, auxiliary transceiver, and switch module. Each transceiver contains T-branch, R-branch, and common branch. The design of each part of the STR is described in this section.

### 4.1. T-Branch

In the assembly process of power amplifier for STR, the final PA-gallium nitride (GaN) power transistor is interconnected with its peripheral circuit through gold wire (gold belt) bonding. Due to the parasitic inductance introduced by the bonding gold wire, the impedance matching of the amplifier is affected [32,33]. In engineering, the effect of parasitic inductance on output matching of PA can be eliminated by adding capacitive debugging blocks (CDB), thus improving the output impedance matching of PA.

Referring to the output power level of the signal from the linear frequency modulation (LFM) source, the output impedance matching design of PA is carried out through tuning the circuit parameters of the inductance–capacitance (L–C) network at the output of GaN power transistor. The tuning methods include:

1. Adjust the relative position between the CDBs and the transmission line.
2. Adjust the arc height and the number of the bonding gold wires which connect the CDBs and the transmission line.
3. Adjust the diameter of the gold wires or the width of the gold belts.
4. Adjust the capacitance values of the DC-block capacitors at input and output of the GaN power transistors.

**Figure 8:**
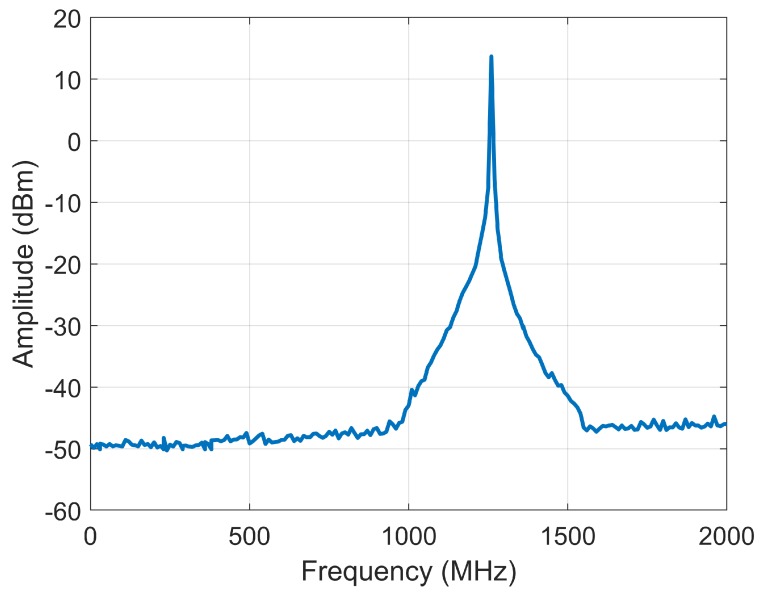
The clutter suppression of the STR in T-mode.

Good performance is obtained in the T-mode of STR. The clutter suppression is improved, which is better than 60 dB, as shown in Figure 8. In addition, the linearity of the phase characteristic is good, the nonlinear phase error of transmitting signal is 3–4 degrees in 150 MHz bandwidth.

### 4.2. R-Branch

The LNA uses the ultra-low noise amplifier which is mainly reactance matching. Reactance matching is an index selective matching, and the index can be the maximum gain, the best noise, or the maximum output power. It is a lossless matching network and a good choice for low noise amplifiers design. Lossy matching is also an effective measure to widen the bandwidth of the LNA. It adds resistance loss to the low-frequency end with higher gain, so as to obtain better flatness. This matching method can achieve good stability, but the NF will deteriorate due to the introduction of resistance loss. Based on the requirements of low noise design, reactance matching is used at the input and lossy matching is used at the output of the transistor to achieve the comprehensive optimization of low noise, broadband, and high stability.

### 4.3. Common Branch

#### 4.3.1. Spatial Interference Suppression Design

An LPF is designed at the antenna port of the circulator in the transceivers. The function of the filter is to filter the spatial interference signal and prevent the interference signal from entering the phase synchronization system through the SANT. S-band is the common frequency band of satellite mobile communication, radio measurement and satellite TT&C. Dozens of satellites in orbit cause serious spectrum congestion. Therefore, filters are needed to filter out the abundant interference signals in this band. Through calculation, an LPF with the insertion loss less than 0.5 dB in 1.15 GHz–1.35 GHz and the stop-band rejection not less than 50 dB in 2 GHz–4 GHz are selected.

#### 4.3.2. Calibration

**Figure 9:**
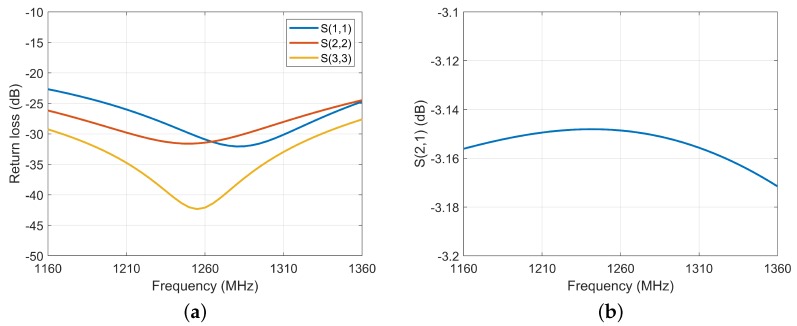
Return loss (**a**) and insertion Loss (**b**) of the two-way WPD.

The calibration circuit consists of a coupler and a power combiner. The calibration signal is generated by coupling the main signal with 30 dB coupling degree through the coupler, and the master and auxiliary calibration signals are synthesized by a power combiner and output to the inner calibrator. Power combination network is used to distribute and synthesize microwave signals. The requirements of this scheme for power division network are as follows: 1. Low insertion loss, good consistency of amplitude and phase of each channel, and the high synthesis efficiency can be guaranteed. 2. The isolation between the two branches is high, so that, when one of the branches fails, it will not affect the normal operation of the other way or have little impact. According to the design requirements, this scheme uses the Wilkinson power divider (WPD). The simulation curves of the two-way WPD for this scheme are shown in Figure 9. The performance of insertion loss and return loss is good.

### 4.4. Switch Module

Four SANTs are designed in the system to obtain omnidirectional coverage. It mainly consists of a double-pole double-throw (DPDT) switch and two single-pole single-throw (SPDT) switches. The DPDT achieves cross backup between the master and auxiliary; two SPDTs achieve selective switching of four SANTs; thus, the master or auxiliary can be connected to any of the four SANTs through the switch module.

Usually, the backup manner of spaceborne circuit includes hot backup and cold backup, and relays electromagnetic, RF coaxial switch, and latching (REMSL) are good choices for cold backup. The advantages of REMSL cold backup include:

1. During the working period, there is only one power-up circuit, and the other one is not power-up, which can effectively reduce the power consumption of the whole machine.
2. Current flows through the switch only at the moment of switching, and the power consumption of switch is zero during normal operation.
3. The reliability of REMSL is high, which makes the reliability of the whole machine remarkably improved.
4. The power loss of the transmitting signal after passing through the switch is only 0.15 dB.
5. The multipaction breakdown power and dissipation breakdown power of the switch are both good, up to 200 W in L band.
6. REMSL is an ultra-wideband device, which can realize a universal design of STR.

**Figure 10:**
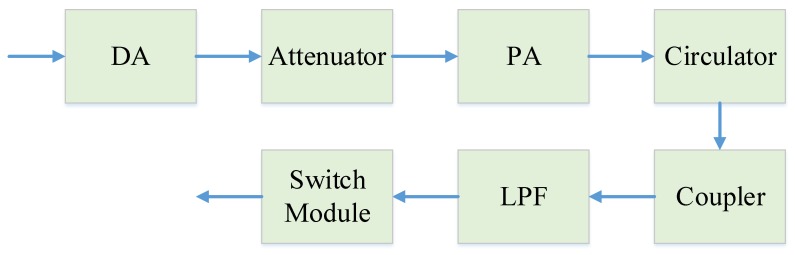
Schematic diagram of the synchronization signal transmitting link.

**Figure 11:**
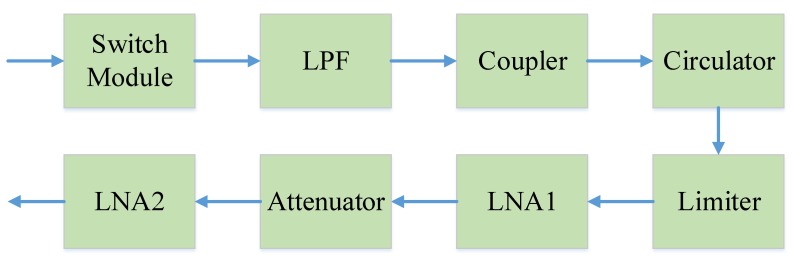
Schematic diagram of the synchronization signal receiving link.

The schematic diagram of the synchronization signal transmitting link of the STR is shown in Figure 10. The schematic diagram of the synchronization signal receiving link of the STR is shown in Figure 11. The formulas of system NF and gain are as follows [34]:

(6) $$\begin{array}{cc} F_{0} & =F_{1}+\frac{F_{2}-1}{G_{1}}+\cdots +\frac{F_{n}-1}{G_{1}G_{2}\cdots G_{n-1}} \end{array}$$

(7) $$\begin{array}{cc} G_{0} & =G_{1}G_{2}\cdots G_{n} \end{array}$$

where $F_{0}$ and $G_{0}$ is the overall NF and gain of a cascaded circuit with *n* (n≥2) elements, $F_{i}$ (1≤i≤n) and $G_{i}$ is the NF and the gain of the *i*th element, respectively. For a passive component with loss $L_{i}$ in ratio, we will have $G_{i}=\frac{1}{L_{i}}$.

The parameters of each part of the circuit in the signal receiving link are brought into the formula. When using the transistor microwave switch scheme, the receiving NF is 4.59 dB and the receiving gain is 24.1 dB. The receiving gain meets the technical requirements of 24–26 dB, but the NF is poor. The detailed calculation process is shown in Table 4.

By adopting REMSL instead of transistor switches, as shown in Table 5, the NF of the receiving link is 3.39 dB, and the receiving gain is 25.3 dB. The receiving gain and NF meet the technical requirements, and the noise figure is optimized by 1.2 dB.

By optimizing the receiving noise figure, SNR of the receiving output signal of the STR is increased, thus the phase error of the intersatellite phase synchronization is further reduced. Therefore, the synchronization accuracy is further improved.

## 5. Test Results and Experimental Verification

### 5.1. Test Results of STR

The prototype of the STR has been developed and participated in a SAR system integration test. The measured performance indexes are shown in Table 6.

**Figure 12:**
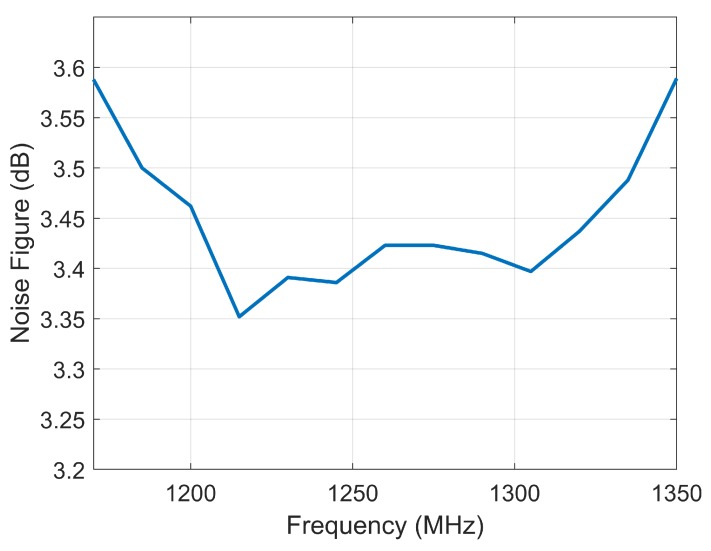
The receiving NF of the STR.

The peak power of transmitting signal is about 38.3 dBm, and the gain of receiving channel is about 25.1 dB. Moreover, good consistency is obtained between the four SANTs in both T-mode and R-mode. The in-band nonlinear phase error is controlled within 3.5 degrees in both T-mode and R-mode, which improves the phase accuracy of the synchronization system. Better noise characteristics are obtained by using the REMSL switch module; as shown in Figure 12, the NF of receiving channel is 3.5 dB in 150 MHz bandwidth. The flatness of amplitude is about 0.9 dB in T-mode and 0.4 dB in R-mode in 150 MHz bandwidth, which is acceptable for the phase synchronization system. In addition, the isolation of switches reaches 65 dB, which can effectively suppress the signal leakage between the SANTs. By comparing the measured parameters and requirements, the STR has good performance. The power consumption, weight, and size of STR are relatively small, which occupy less resources on the satellite.

### 5.2. Accuracy Analysis

**Figure 13:**
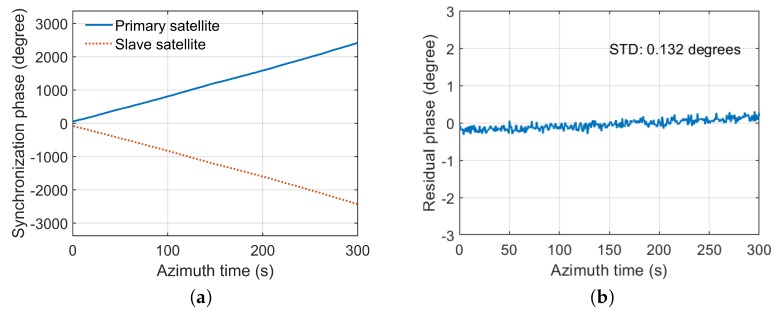
The test results with 61 dB SNR. (**a**) the synchronization phases of two satellites; (**b**) the residual phase after compensation.

The accuracy of phase synchronization is evaluated to verify the effectiveness of STR. In the first experiment, the SNR of synchronization is 61 dB. The synchronization phases of primary and slave satellite are shown in Figure 13a. An optical delay line is used to simulate the echo receiving process for the two satellites. The primary satellite transmits the radar signal. The radar signal passes through the optical delay line and then is received by the primary satellite and slave satellite. After range compression, the peak phase $\varphi_{p}$ and $\varphi_{s}$ of the radar echo signal are extracted in the peak position for primary satellite and slave satellite, respectively. Therefore, $\varphi_{s}-\varphi_{p}$ can be regarded as the reference phase to evaluate the synchronization accuracy. The residual phase in the test experiment can be written as

(8) $$\varphi_{res}=\varphi_{c}-\left(\varphi_{s}-\varphi_{p}\right)$$

**Figure 14:**
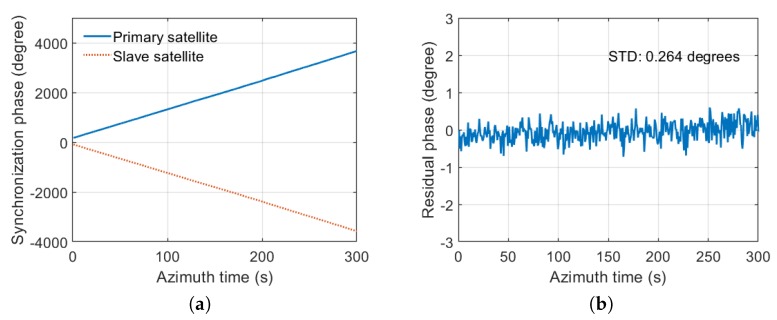
The test results with 42 dB SNR. (**a**) the synchronization phases of two satellites; (**b**) the residual phase after compensation.

The synchronization phase is extracted in the peak position of compressed synchronization signal after pulse compression. The synchronization phase $\varphi_{12}\left(t\right)$(Primary satellite) and $\varphi_{21}\left(t\right)$(Slave satellite) are shown in Figure 13a. After compensation, the residual phase $\varphi_{res}$ is shown in Figure 13b. The STD of the residual phase is 0.132 degrees. In the second experiment, the SNR of synchronization is 42 dB. The test results are shown in Figure 14. The STD of the residual phase is 0.264 degrees. The STD of the residual phase is less than 0.3 degrees, which proves that the synchronization scheme has high synchronization accuracy.

## 6. Conclusions

In BiSAR systems, the phase synchronization is an important issue that must be addressed. An advanced non-interrupted phase synchronization scheme is used for LuTan-1. Both satellites are equipped with four SANTs for a mutual exchange of synchronization signals, which are exchanged immediately after the ending time of the radar echo-receiving window and before the starting time of the next PRI. STR is a novel unit in the electronics subsystem, which can be used for transmitting and receiving the synchronization signal. The function and working principle of STR are introduced in this paper, and the structure of STR is also described in detail. The STR contains master, auxiliary transceivers, and switch modules. Moreover, each transceiver contains a T-branch, R-branch, and common branch. The performance of each part in STR is evaluated, and the measured results prove that the STR has good performance. The STD of the residual phase is less than 0.3 degrees, which prove that the synchronization scheme has high synchronization accuracy. The results have guiding significance for the synchronization unit design of LuTan-1 and the future BiSAR system. In the future, further tests will be carried out to verify the stability of the STR. 

## Figures and Tables

**Table 1:** Main parameters of LuTan-1.

Parameter	Value
Orbit height (km)	607
Carrier frequency (GHz)	1.26
Channel number	4
Maximum synchronization frequency	PRF/2
Polarization	quad/compact/single

**Table 2:** An Example of Beam for LuTan-1.

Parameter	Value
Swath width (km)	50
Resolution (m)	3 × 3
Look angle (deg.)	18–22.40
Radar pulse width (μs)	110
Echo receiving window width (μs)	250
Synchronization pulse width (μs)	10
PRF (Hz)	1723
Maximum synchronization frequency (Hz)	861.5

**Table 3:** The main technical requirements of the STR.

Parameter	Requirement
Operation bandwidth (MHz)	≥150
Peak power of transmitting signal (dBm)	37–39
In-band flatness of amplitude of transmitting signal (dB)	≤1
In-band nonlinear phase error of transmitting signal (deg.)	≤5
Noise figure (NF) of receiving channel (dB)	≤3.7
Gain of receiving channel (dB)	24–26
In-band flatness of amplitude of receiving signal (dB)	≤0.5
In-band nonlinear phase error of receiving signal (deg.)	≤5
Isolation of switches (dB)	≥60
Power consumption (W)	≤4.0
Weight (kg)	≤3.5
Size (mm^3^)	≤200 × 239 × 91

**Table 4:** The NF of receiving link with transistor microwave switches.

Device	Microwave Switch	LPF & Coupler	Circulator & Limiter	LNA1	Fixed Attenuator	LNA2
Gain (dB)	−1.6	−0.7	−1.4	18.5	−3	12.3
NF (dB)	1.6	0.7	1.4	0.6	3	5.2
System gain (dB)	−1.6	−2.3	−3.7	14.8	11.8	24.1
System NF (dB)	1.6	2.3	3.7	4.3	4.35	4.59

**Table 5:** The NF of receiving link with REMSL.

Device	Microwave Switch	LPF & Coupler	Circulator & Limiter	LNA1	Fixed Attenuator	LNA2
Gain (dB)	−0.4	−0.7	−1.4	18.5	−3	12.3
NF (dB)	0.4	0.7	1.4	0.6	3	5.2
System gain (dB)	−0.4	−1.1	−2.5	16	13	25.3
System NF (dB)	0.4	1.1	2.50	3.10	3.15	3.39

**Table 6:** The measured results of the STR.

Parameter	SANT1	SANT2	SANT3	SANT4
Peak power of transmitting signal (dBm)	37.9–38.8	37.9–38.8	37.9–38.8	37.9–38.8
In-band flatness of amplitude of transmitting signal (dB)	0.89	0.83	0.91	0.88
In-band nonlinear phase error of transmitting signal (deg.)	3.5	3.5	3.4	3.5
NF of receiving channel (dB)	3.5	3.5	3.5	3.5
Gain of receiving channel (dB)	24.9–25.3	24.9–25.3	24.9–25.3	24.9–25.3
In-band flatness of amplitude of receiving signal (dB)	0.4	0.4	0.4	0.4
In-band nonlinear phase error of receiving signal (deg.)	3.2	3.2	3.2	3.2
Isolation of switches (dB)	65.0	65.0	65.0	65.0
Power consumption (W)	3.9			
Weight (kg)	3.45			
Size (mm^3^)	199.6×238.7×90.7			

## Abbreviations

The following abbreviations are used in this manuscript:

SAR	Synthetic aperture radar
BiSAR	Bistatic synthetic aperture radar
PRF	Pulse-repetition frequency
PRI	Pulse-repetition internal
STR	Synchronization transceiver
PA	Power amplifier
LPF	Low-pass filter
LNA	Low-noise amplifier
GaN	Gallium nitride
SNR	Signal-to-noise ratio
LFM	Linear frequency modulation
SANT	Synchronization antenna
NF	Noise figure
CDB	Capacitive debugging blocks
WPD	Wilkinson power divider
DPDT	Double-pole double-throw
SPDT	Single-pole single-throw
REMSL	Relays electromagnetic, RF coaxial switch, latching
TT&C	Telemetry, tracking, and command
T-mode	Transmitting mode
R-mode	Receiving mode
STD	Standard deviation
